# A connexin30 mutation rescues hearing and reveals roles for gap junctions in cochlear amplification and micromechanics

**DOI:** 10.1038/ncomms14530

**Published:** 2017-02-21

**Authors:** Victoria A. Lukashkina, Snezana Levic, Andrei N. Lukashkin, Nicola Strenzke, Ian J. Russell

**Affiliations:** 1 Sensory Neuroscience Research Group, School of Pharmacy and Biomolecular Sciences, University of Brighton, Brighton BN2 4GJ, UK; 2 Brighton and Sussex Medical School, University of Sussex, Brighton BN1 9PX, UK; 3 Department of Otorhinolaryngology, University Medicine Göttingen, Robert-Koch-Strasse 40, Göttingen 37075, Germany

## Abstract

Accelerated age-related hearing loss disrupts high-frequency hearing in inbred CD-1 mice. The p.Ala88Val (A88V) mutation in the gene coding for the gap-junction protein connexin30 (Cx30) protects the cochlear basal turn of adult CD-1*Cx30*^*A88V/A88V*^ mice from degeneration and rescues hearing. Here we report that the passive compliance of the cochlear partition and active frequency tuning of the basilar membrane are enhanced in the cochleae of CD-1*Cx30*^*A88V/A88V*^ compared to CBA/J mice with sensitive high-frequency hearing, suggesting that gap junctions contribute to passive cochlear mechanics and energy distribution in the active cochlea. Surprisingly, the endocochlear potential that drives mechanoelectrical transduction currents in outer hair cells and hence cochlear amplification is greatly reduced in CD-1*Cx30*^*A88V/A88V*^ mice. Yet, the saturating amplitudes of cochlear microphonic potentials in CD-1*Cx30*^*A88V/A88V*^ and CBA/J mice are comparable. Although not conclusive, these results are compatible with the proposal that transmembrane potentials, determined mainly by extracellular potentials, drive somatic electromotility of outer hair cells.

## Abstract (web summary)

A point mutation in the gap-junction protein connexin 30 stops early onset age-related hearing loss. Here, the authors show that gap junctions contribute to cochlear micromechanics and that cochlear amplification is likely controlled by extracellular potentials in vicinity of the cochlear sensory cells.

Responses recorded from the cochleae of wild-type (WT) mice are very sensitive and sharply tuned with a frequency range that extends from ∼2 kHz to above 100 kHz (ref. 1). These characteristics depend on the inherent mechanical properties of the basilar membrane (BM), which is graded in increasing stiffness from the apex to the base of the cochlea^2^. Signal processing in the cochlea is initiated when sound-induced changes in fluid pressure displace the BM in the transverse direction, causing radial shearing displacements between the surface of the organ of Corti (OC; the reticular lamina) and the overlying tectorial membrane (TM; Fig. 1a)^3^. The stereocilia on the apical surface of outer hair cells (OHCs) provide an elastic link between the OC and the overlying TM^4^. Deflection of the stereocilia by the radial shear^5^ gates the hair cell's mechanoelectrical transducer (MET) channels, thereby initiating a MET current^6^ that promotes active mechanical force production by the OHCs, which, in turn, influences mechanical interactions between the TM and the BM^7^,^8^. This nonlinear frequency-dependent enhancement process, which boosts the sensitivity of cochlear responses to low-level sounds and compresses them at high levels, is known as the cochlear amplifier^9^.

These characteristics are shared by all normal-hearing mouse strains, but can be lost with age, initially from the basal, high-frequency regions of the cochlea. High-frequency hearing in the CD-1 mouse deteriorates progressively from about 3 weeks in age^10^. Pathological changes in cochlear fibrocytes, especially in the spiral ligament, precede other presbycusic changes associated with age-related hearing loss (ARHL) in the CD-1 mouse^10^. These fibrocytes, like many cell types in the cochlea, are coupled together by intercellular gap junctions. Each gap junction is formed by two interacting hemichannels (connexons) on neighbouring cells, each consisting of six connexin protein subunits, to permit the bidirectional flow of ions and signalling molecules. The hemichannels of type 1 fibrocytes of the spiral ligament, supporting cells of the sensory epithelium of the cochlea, the OC and cells within the basal cell region of the stria vascularis (SV) are formed of co-localized Cx26 and Cx30 (ref. 11), deletions or mutations of which are responsible for the majority of genetically based hearing loss^12^. Mutations of Cx30, including A88V (ref. 13), are the basis for Clouston syndrome (OMIM #129500), an autosomal dominant genetic disorder characterized by alopecia, nail dystrophies, palmoplantar hyperkeratosis and sometimes hearing loss. The CD-1*Cx30*^*A88V/A88V*^ mouse model carrying the p.Ala88Val (A88V in NP_001010937.1) point mutation of Cx30 was generated by Bosen *et al*.^13^ primarily to analyse the skin phenotype that expresses many of the phenotypes of Clouston syndrome. Surprisingly, in addition to mild low-frequency hearing loss, the A88V mutation led to rescue of the high-frequency hearing loss expressed in the CD-1 background strain^13^. Here we confirmed this finding and also discovered that active frequency tuning of the BM and apparent passive compliance of cochlear partition are enhanced in the cochleae of CD-1*Cx30*^*A88V/A88V*^ mice compared to CBA/J mice with sensitive high-frequency hearing. CD-1*Cx30*^*A88V/A88V*^ mice preserve excellent sensitivity in their basal cochleae and normal saturating amplitudes of the cochlear microphonic (CM) in spite of the fact that they have a greatly reduced endocochlear potential (EP). We suggest that somatic electromotility depends on OHC transmembrane potential difference due primarily to extracellular potential changes in the vicinity of the OHCs rather than on OHC intracellular potentials as originally proposed by Dallos and Evans^14^.

## Results

### Cx30 similarly located in CD-1*Cx30* ^*A88V/A88V*^ and CBA/J mice

**Figure 1:**
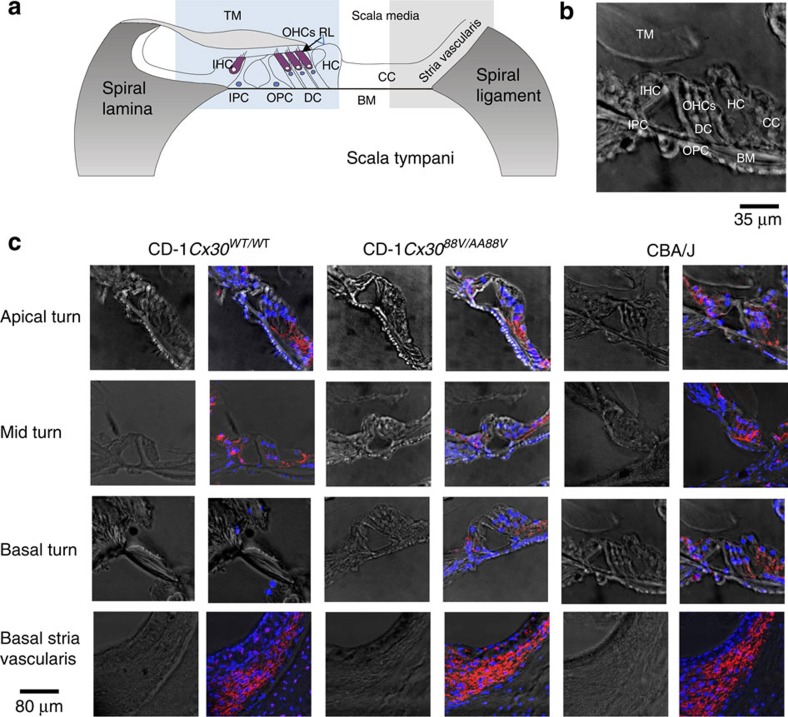
Cx30 immunoreactivity in cochleae of CBA/J and CD-1*Cx30*^^*A88V/A88V*^^ mice are similar. (**a**) Schematic cross-section of the cochlea showing cells of the sensory epithelium (organ of Corti) including inner pillar cells (IPCs), outer pillar cells (OPCs), Deiters' cells (DCs), Hensen cell (HC), outer hair cell (OHC), inner hair cell (IHC), Claudius cells (CCs) and major non-cellular elements (basilar membrane (BM), tectorial membrane (TM) and reticular laminar (RL); modified with permission from Fig. 1 (ref. 49). (**b**) Confocal micrograph of 10 μm cryosection taken from middle turn of cochlea in **c** to identify details of cells and noncellular structures in the organ of Corti. Modified from **c**. Rows of confocal micrographs of 10 μm cryosections of the apical, middle and basal turns of the organ of Corti and stria vascularis. The rows are organized in columns of pairs of micrographs at each location from *CD-1Cx30*^*WT/WT*^, CD-1*Cx30*^*A88V/A88V*^ and CBA/J mice. The left of each pair of micrographs shows the unstained section. In the right of each pair, the Cx30 (red) expression is revealed with a selective antibody and nuclei are counterstained with DAPI (blue). OHCs, DCs and spiral lamina cells are intact in all cochlea turns of CBA/J and CD-1*Cx30*^*A88V/A88V*^ mice but not in the basal turn of CD-1*Cx30*^*WT/WT*^ mice. Cx30 appears to be localized in the membranes in basal cells of the stria vascularis, DCs, IPCs, OPCs and spiral lamina cells of the intact OC, that is, in all turns of the cochleae of CBA/J and CD-1*Cx30*^*A88V/A88V*^ mice. Scale Bar, 35 μm (**b**) and 80 μm for all micrographs in **c**, see blue and grey squares in **a** for location of histology shown in rows 1–3 (organ of Corti) and 4 (stria vascularis), respectively. All mice were 3 months old.

According to the histology and Cx30 immunohistochemistry, the OC is structurally intact in all turns of the cochleae of CBA/J (*n*=4) and CD-1*Cx30*^*A88V/A88V*^ (*n*=7). In contrast, the basal, high-frequency turn of CD-1*Cx30*^*WT/WT*^ (*n*=7) mice is degenerated, with total loss of OHCs (Fig. 1c). In intact turns of the cochlea (CBA/J and CD-1*Cx30*^*A88V/A88V*^), Cx30 is localized in the membranes of Deiters' cells (DC) and outer pillar cells (OPCs) in the OC and in basal cells of the SV and spiral ligament (Fig. 1).

### CD-1*Cx30* ^*A88V/A88V*^ mice generate high-frequency DPOAEs

Distortion product otoacoustic emissions (DPOAEs) are nonlinear acoustical responses of the cochlea recorded in the ear canal in response to simultaneous stimulation with two pure tones f1 and f2. DPOAEs are usually dominated by the cubic distortion product at frequency 2f1–f2. DPOAEs are consequences of the nonlinear properties of cochlear mechanosensory transduction mechanisms^15^ and cochlear amplification^16^. Therefore, DPOAE threshold as a function of f2 stimulus frequency provides information about the sensitivity and frequency range of cochlear responses at the level of the OHCs, which is the focus of interest in this study.

**Figure 2:**
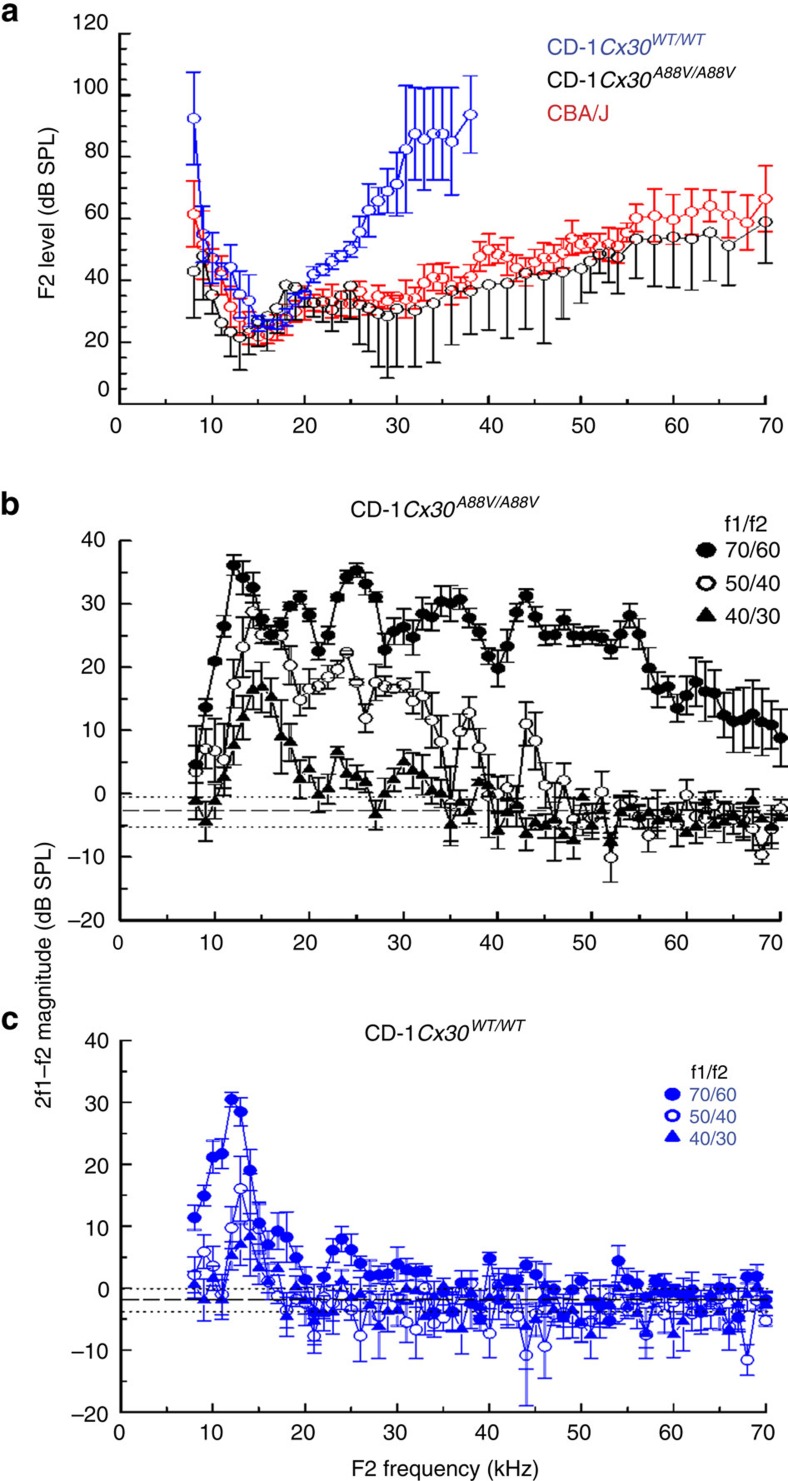
DPOAE audiograms. (**a**) DPOAE threshold (2f1–f2, 0 dB SPL threshold criterion, mean±s.d.) as a function of the f2 frequency (f2/f1 ratio=1.23; level of f2 set 10 dB below f1 level) from six CD-1*Cx30*^*WT/WT*^ (blue symbols) and five CD-1*Cx30*^*A88V/A88V*^ (black symbols) mice, and from seven CBA/J mice (red symbols). The maximum SPL of the sound system was restricted to ≤110 dB for frequencies ≥35 kHz. (**b**,**c**) DPOAE magnitude (2f1–f2, mean±s.d.) as a function of the f2 frequency for five CD-1*Cx30*^*A88V/A88V*^ mice (**b**) and five CD-1*Cx30*^*WT/WT*^ mice (**c**). Stimulus levels and colour and symbol coding for f1 and f2 are shown in the figures. *X* axis: f2 frequency (kHz) for all figures. Dashed and dotted lines indicate the recording noise floor±s.d. for all measurements.

Bosen *et al*.^13^ demonstrated that for frequencies <10 kHz the thresholds for auditory brainstem responses (ABRs) recorded from CD-1*Cx30*^*A88V/A88V*^ mice are increased compared to those from their CD-1*Cx30*^*WT/WT*^ littermates. However, the thresholds of both ABRs and DPOAEs of CD-1*Cx30*^*A88V/A88V*^ mice are decreased and their amplitudes increased significantly compared to those of CD-1*Cx30*^*WT/WT*^ littermates for frequencies above 16 kHz. Within the sensitivity range of the high-frequency sound system used in our measurements, DPOAE threshold audiograms (Fig. 2a) recorded from CD-1*Cx30*^*A88V/A88V*^, CD-1*Cx30*^*WT/WT*^, CBA/J mice are similar for frequencies below 20 kHz. Above 20 kHz, the audiograms of the CD-1*Cx30*^*WT/WT*^ become less sensitive with increasing frequency. These characteristics may also be observed in the DPOAE magnitudes as functions of f2 frequency recorded from individual CD-1*Cx30*^*A88V/A88V*^ (Fig. 2b) and CD-1*Cx30*^*WT/WT*^ (Fig. 2c) mice. The DPOAE audiograms of CD-1*Cx30*^*A88V/A88V*^ and CBA/J mice are closely similar and reveal that OHC-mediated mechanical sensitivities of the OCs of CD-1*Cx30*^*A88V/A88V*^ and CBA/J mice extend at least to the 70 kHz frequency range. Thus, the DPOAE measurements reported here accord with those reported previously^13^ and further reveal that cochlear sensitivity is preserved across the entire basal turn of the CD-1*Cx30*^*A88V/A88V*^ mouse cochlea.

### Reduced EP in CD-1*Cx30* ^*A88V/A88V*^ and Cx30^A88V/WT^ mice

The EP is generated by electrogenic secretion of potassium-rich endolymph from the SV^17^. EP augments the mechanoelectrical transduction (MET) current, hence cochlear amplification. The latter is due to forces produced by prestin-based, voltage-dependent, somatic motility of OHCs^18^,^19^,^20^,^21^,^22^,^23^,^24^,^25^ and perhaps hair bundle motility^26^, which amplify sound-induced BM vibrations^24^. Reduction of the EP impairs the sensitivity and frequency tuning of cochlear responses^27^,^28^,^29^.

EP was measured in the scala media by advancing the micropipettes through the OC. The EP, expressed as mean±s.d. measured from CD-1*Cx30*^*WT/WT*^ mice was +112.8±1.2 mV, *n*=9, not significantly different from that measured from CBA/J mice of a similar age (+114.7±2.9 mV, *n*=4; *p*=0.11, unpaired two-tailed *t*-test). In contrast, EP was greatly reduced to +88.4±2.0 mV in CD-1*Cx30*^*A88V/WT*^ mice (*n*=8) and to only +71.3±2.8 mV (*n*=12) in CD-1*Cx30*^*A88V/A88V*^ littermates (*p*<0.0001 for CD-1*Cx30*^*WT/WT*^ compared to CD-1*Cx30*^*A88V/WT*^ or CD-1*Cx30*^*A88V/A88V*^ mice; *p*=0.0016 for CD-1*Cx30*^*A88V/WT*^ versus CD-1*Cx30*^*A88V/A88V*^, unpaired *t*-test). Hence, a higher expression of mutated Cx30 A88V protein subunits appears to entail a greater reduction in EP.

### CD-1*Cx30^A88V/A88V^* mice produce CM

**Figure 3:**
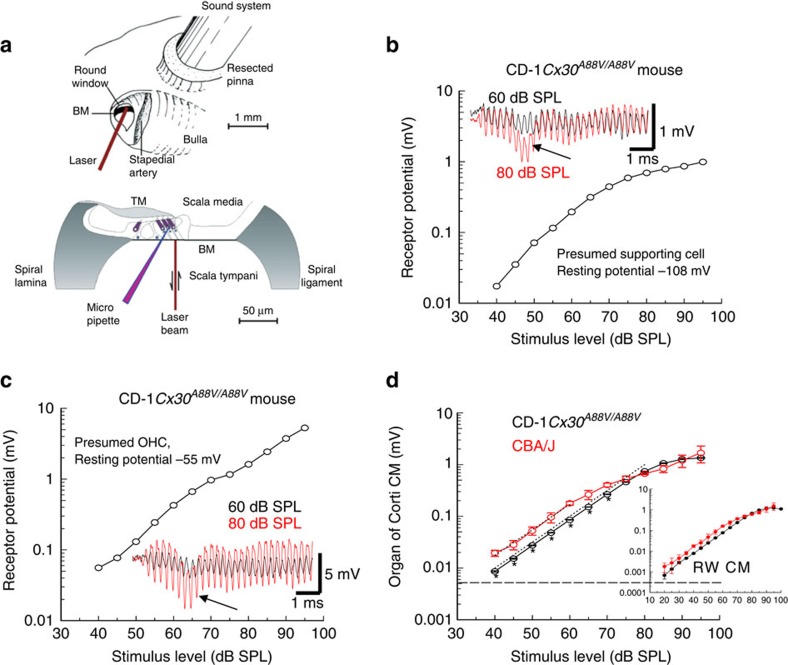
Magnitude of receptor potential and CM as functions of stimulus level. Recordings from the basal turn cochleae of CD-1*Cx30*^*A88V/A88V*^ and CBA/J mice in response to 5 kHz tones. (**a**) Techniques (in addition to those presented in Fig. 2) used to make acoustic, electrophysiological and mechanical measurements from the cochlea (modified with permission from refs 39, 40). (**b**) Peak-to-peak magnitude of an intracellular receptor potential recorded from a presumed supporting cell from a CD-1*Cx30*^*A88V/A88V*^ mouse in response to a 5 kHz tone as a function of stimulus level (representative example). (**c**) Magnitude of an intracellular receptor potential of a presumed OHC of a CD-1*Cx30*^*A88V/A88V*^ mouse in response to a 5 kHz tone as a function of stimulus level (representative example). Insets in **b** and **c** show voltage responses to 5 kHz tones made at tone onset; stimulus levels: 60 dB SPL, black; 80 dB SPL, red. Arrows, insets of **b** and **c** indicate negative peak of presumed compound action potential. (**d**) Compound extracellular receptor potentials (organ of Corti CM), as functions of stimulus level to 5 kHz tones, measured close to the middle row of OHCs (mean±s.d.) from cochleae of CBA/J (red, *n*=5) mice and CD-1*Cx30*^*A88V/A88V*^ (black, *n*=4) mice. Asterisks: significantly different (unpaired *t*-test, ≤0.05 two-tailed *p* value). Dashed line: recording noise floor for **d** and inset; CM responses were not seen at any level above this floor for CD-1*Cx30*^*A88V/WT*^ and CD-1*Cx30*^*WT/WT*^ mice. Dotted lines: slope of one. Inset, CM recorded from the round windows of the same group of mice (RW CM).

Cochlear amplification is initiated by the flow of MET current through channels located at the tips of the stereocilia that comprise the OHC hair bundles^30^. The driving force for this K^+^-dominated current is provided by batteries in series: the resting membrane potential (approximately −50 mV for OHCs^31^,^32^) and the EP (approximately +120 mV in mice)^3^,^33^,^34^. The total OHC MET current flow across the total electrical impedance of cochlear partition can be monitored by measuring the CM potential. These extracellular potentials, which can be recorded at the round window (RW), are dominated by basal turn OHC MET currents^35^,^36^. In this study, we did not use CM to assess cochlear amplification, sensitivity or frequency selectivity, but to assess mechanoelectrical transduction of OHCs in the basal turn. We therefore stimulated the ear with 5 kHz tones, which is far below the 50–80 kHz frequency range of the basal turn cochlear responses. We chose this frequency because the entire basal turn of the cochlea should be displaced in unison^22^ and at saturating levels of the CM, all OHCs in the basal turn of the cochlea will contribute MET current to the CM^34^,^35^. Stimulation with high-frequency tones close to the sensitive frequency range of the basal turn will cause adjacent regions of the cochlear partition of the basal turn to move in opposite directions^22^, thereby causing complex phase augmentation and cancellation of the CM^35^, which defeats the purpose of the measurement, which is simply to compare the functionality of mechanoelectrical transduction in basal turn OHCs from CD-1*Cx30*^*A88V/A88V*^, CD-1*Cx30*^*WT/WT*^ and CD-1*Cx30*^*A88V/WT*^ littermates with that recorded from control CBA/J mice. Any damage to or loss of OHCs will be indicated as a reduction in CM (see Methods), or indeed lack of detectable CM in the case of total absence of functional OHCs in the basal turn of the cochlea. Consistent with our histological findings, we recorded CM potentials from the OC (Fig. 3d) and RW (Fig. 3d, inset), only from CD-1*Cx30*^*A88V/A88V*^ mice. CM was not detectable in CD-1*Cx30*^*WT/WT*^ and CD-1*Cx30*^*A88V/WT*^ littermates (not shown).

We confirmed our RW recordings from CD-1*Cx30*^*A88V/A88V*^ mice with sharp quartz glass micropipettes advanced through the RW and BM of the basal turn of the cochlea and into the OC towards the scala media (Fig. 3a). With this approach, we recorded receptor potentials from putative supporting cells with very negative resting potentials (−108±0.9 mV, *n*=24; mean±s.d., number of cells, Fig. 3b). Larger receptor potentials in response to 5 kHz tones were recorded from cells with smaller resting potentials (−50.6±2.0 mV, *n*=8, Fig. 3c) that were encountered just before penetrating into the scala media. These cells were tentatively identified as OHCs in that they share the characteristics described previously for OHCs in the basal turn of the guinea pig cochlea^37^. In contrast to intracellular measurements from cells in the guinea pig OC^38^, we observed a transient hyperpolarizing dip occurring ∼2 ms after tone onset in stable (>5 min duration) intracellular voltage responses recorded from presumed supporting cells and OHCs in the basal turn of the mouse cochlea (arrows in insets of Fig. 3b,c). As its latency is compatible with a combination of travelling wave and synaptic delays it is likely that the transient hyperpolarization represents the compound action potential of the 8th nerve. The potential is also present in extracellular spaces of the OC (Fig. 3d). It would appear that the intracellular recordings from presumed mouse OHCs are electrically more leaky than those made from the guinea pig cochlea, where intracellular recordings of neural potentials have not been seen^36^,^37^. It is possible, through differential subtraction across the hair cell membranes, that this potential does not influence the electrical responses of the cells within the OC as has been suggested for other extracellular potentials in measurements from the guinea pig cochlea^38^. Significantly, the peak-to-peak magnitude of the CM recorded from the extracellular spaces close to the OHCs (Fig. 3d) or from the RW (inset of Fig. 3d) of normal-hearing CBA/J mice, and CD-1*Cx30*^*A88V/A88V*^ mice are very similar for stimulus levels above 75 dB SPL. For stimulus levels between 40 and 60 dB SPL, the magnitudes of CM recorded from the CBA/J and CD-1*Cx30*^*A88V/A88V*^ mice both increase with increasing stimulus level with a slope of 1.12 (close to 1, Fig. 3d, dotted line). However, the amplitude of CM measured from CD-1*Cx30*^*A88V/A88V*^ mice for any given stimulus level below ∼60 dB SPL, is only 45% of that recorded from CBA/J mice (Fig. 3d).

### Sharp sensitive BM tuning in CD-1*Cx30* ^*A88V/A88V*^ mice

**Figure 4:**
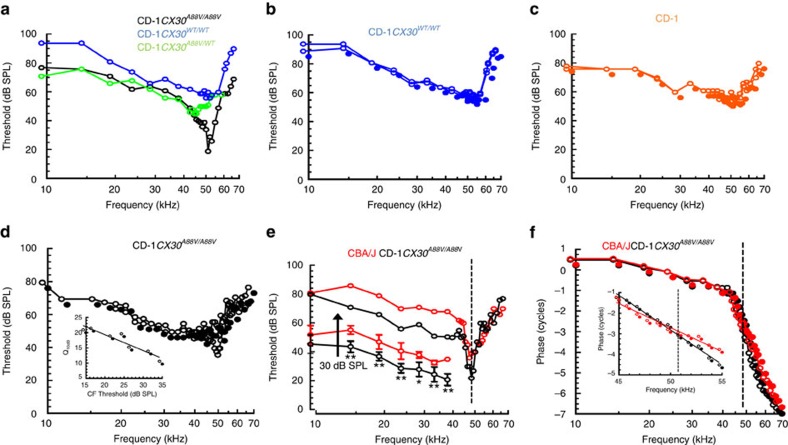
Basilar membrane displacement frequency tuning curves and phase. (**a**–**e**) BM displacement threshold (0.2 nm criterion) as a function of stimulus frequency measured from the basal turn of the cochlea. Strain and genetic state of individual mice identified in the wording and colour of the captions at the top of each figure (CD-1*Cx30*^*A88V/A88V*^ (black), CD-1*Cx30*^*WT/WT*^ (blue), CD-1*Cx30*^*A88V/WT*^ (green), CD-1 (orange) CBA/J (red)). Curves with solid symbols in **b**–**d** are post-mortem measurements from the same preparations. Inset to **d**: Q_10 dB_ as a function of threshold at the tuning curve tip for CD-1*Cx30*^*A88V/A88V*^ mice. (**e**) Inset to **e**: mean±s.d., *n*=5 of the 10–40 kHz region of tuning curves for CBA/J and CD-1*Cx30*^*A88V/A88V*^. For clarity, the curves are displaced downwards by 30 dB SPL. Asterisks: significantly different (unpaired *t*-test, **0.01, *0.05 two-tailed *p* value). (**f**) Phase of BM responses (measured at 70 dB SPL) (open symbols) as a function of frequency for tuning curves shown in **e**. Solid symbols, mean±s.d. of phase (10–45 kHz) from five each of CD-1*Cx30*^*A88V/A88V*^ and CBA/J mice. Inset to **f**: linear plots of expanded section of curves between 45 and 55 Hz. The slopes of regression reveal that the phase of BM responses from the CBA/J mouse decreases by −0.232±s.e. 0.014 cycles per kHz and that from the CD-1*Cx30*^*A88V/A88V*^ mouse decreases by −0.335±s.e. 0.011 cycles per kHz.

The ability to resolve sound into individual frequency components depends on BM frequency tuning^29^. BM displacement threshold frequency tuning curves (0.2 nm criteria) were measured from the cochleae of five CD-1*Cx30*^*WT/WT*^ mice, five CD-1*Cx30*^*A88V/WT*^ mice, eight CD-1*Cx30*^*A88V/A88V*^ mice, five CD-1 as controls for the background of the CD-1*Cx30*^*A88V/A88V*^ mice, and four CBA/J mice as examples of mice with excellent hearing and without early onset ARHL. A laser diode self-mixing interferometer was focused through the RW membrane onto locations one third across the width (coincident with outer pillar cells—row 1 OHCs) of the basal turn BM from its attachment to the spiral lamina (Fig. 3a). In this location, which corresponded to the 50–56 kHz region of the BM, magnitude and phase of BM displacement was measured in response to pure tones. BM displacement threshold frequency tuning curves of CD-1*Cx30*^*A88V/WT*^ (Fig. 4a) and CD-1*Cx30*^*WT/WT*^ (Fig. 4a,b) mice are similar to those of CD-1 mice (Fig. 4c) with broad, insensitive minima in the 45–55 kHz range. Post mortem, responses are mostly unchanged (Fig. 4b,c). Thus, in support of the immunohistochemistry and CM measurements, it appears there are no functional OHCs in the basal turn of CD-1*Cx30*^*WT/WT*^ and CD-1*Cx30*^*A88V/WT*^ littermates and CD-1 strain mice.

Examples of BM displacement threshold frequency tuning curves for CD-1*Cx30*^*A88V/A88V*^ mice measured from the 50–56 kHz region of the BM are shown in Fig. 4a,d,e. For comparison, Fig. 4e shows data from a CBA/J mouse with a CF that coincides with the CF of the CD-1*Cx30*^*A88V/A88V*^ frequency tuning curve. Thresholds and frequency tuning in CBA/J mice was very well comparable with published data sets from WT *Otoa*^*EGFP/EGFP*^ (ref. 39) and WT *Tectb*^40^ mice. In contrast, the thresholds of the tuning curves measured from CD-1*Cx30*^*A88V/A88V*^ mice are 22.7±5.8 dB SPL (*n*=8). These were not significantly different from the thresholds measured in CBA/J mice (24.8±3.7 dB SPL, *n*=4, *p*=0.78, two-tailed unpaired *t*-test). In contrast, the bandwidths of the tuning curves measured from CD-1*Cx30*^*A88V/A88V*^ mice were significantly narrower than those of WT mice: the Q_10 dB_ value (characteristic frequency/bandwidth 10 dB from tip) of CD-1*Cx30*^*A88V/A88V*^ was 17.4±3.1 (mean±s.d.) compared with 8.7±4.3 for CBA/J mice (*p*=0.0023, two-tailed unpaired *t*-test). The high- and low-frequency slopes of BM tuning curves, measured from the tip, to 20 dB above the tip, from CD-1*Cx30*^*A88V/A88V*^ mice were 147±8 and 322±15 dB per octave, respectively, which is significantly steeper than in CBA/J mice of 99±6 and 187±11 dB per octave (*p*<0.0001 for high- and low-frequency slopes, two-tailed unpaired *t*-test). Q_10 dB_ of CD-1*Cx30*^*A88V/A88V*^ mice was correlated (*r*=−0.975) with the sensitivity at the tip of the threshold tuning curve; the more sensitive the preparation, the sharper the tuning (Fig. 4d, inset). In line with our interpretation that the sharp amplified tip of the threshold curves for *Cx30*^*A88V/A88V*^ mice derives from active processes, the sensitivity of post-mortem BM tuning curves of CD-1*Cx30*^*A88V/A88V*^ mice (Fig. 4d) resembled those of CD-1*Cx30*^*WT/WT*^ mice (Fig. 4a,b).

The phase of BM responses as functions of stimulus frequency (relative to that of the malleus) were measured from CD-1*Cx30*^*A88V/A88V*^ and CBA/J mice (Fig. 4f) with a common CF (Fig. 4e) at stimulus levels where the BM mechanics are dominated by its passive behaviour (70 dB SPL). The phase-frequency relationships of the CD-1*Cx30*^*A88V/A88V*^ and CBA/J mice are similar in the low-frequency tail region. However, for frequencies in the range of 45–55 kHz, the phase-frequency relations of the CD-1*Cx30*^*A88V/A88V*^ mouse are steeper than those of the CBA/J mouse (Fig. 4f, inset and caption), which may indicate that gap junctions contribute to energy distribution in the active cochlea resulting in the observed sharper frequency tuning of CD-1*Cx30*^*A88V/A88V*^ mice.

### Enhanced passive BM mechanics in CD-1*Cx30* ^*A88V/A88V*^ mice

It is generally accepted that for the tail frequencies of the BM displacement threshold frequency tuning curves the BM response is dominated by stiffness of cochlear partition at a given cochlear location^41^. Thresholds of the tails between 15 and 40 kHz were significantly more sensitive (Fig. 4e, inset) in CD-1*Cx30*^*A88V/A88V*^ mice than in CBA/J mice by 11.0±0.8 dB SPL (*n*=5). No significant difference could be observed at 10 kHz, which we attribute to the large noise floor, which made measurements difficult. We were unable to detect a significant difference in the phase of BM displacement in the tails of the low-frequency tuning curves in the 10–45 kHz region (expressed as mean±s.d., *n*=5, Fig. 4f). The sensitivities of the low-frequency tails of CBA/J, CD-1 and CD-1*Cx30*^*WT/WT*^ mice are similar (not shown but can be deduced from Fig. 4a–c,e), while the sensitivities of the low-frequency tails of tuning curves from CD-1*Cx30*^*A88V/WT*^ mice (not shown) are more variable and less sensitive than those of CD-1*Cx30*^*A88V/A88V*^ mice by 3.2±1.6 dB SPL. It is likely that the gap junctions contribute to the passive stiffness of the cochlear partition because increased sensitivity of the low-frequency tail in CD-1*Cx30*^*A88V/A88V*^ mice and, hence, decreased mechanical stiffness of the cochlear partition persisted post mortem.

## Discussion

If, as generally accepted, MET current flow is controlled by EP in series with the hair cell resting potential^3^,^34^, it is remarkable that DPOAE audiograms and BM sensitivity in the basal turn of CD-1*Cx30*^*A88V/A88V*^ mice are similar to those of CBA/J and other WT mice with excellent hearing^42^,^43^,^44^,^45^. Indeed, as a consequence of the reduced EP, the driving force for MET current flow through the OHC hair bundles in CD-1*Cx30*^*A88V/A88V*^ mice should be reduced to 73% of the CBA/J mouse values (EP_*Cx30A88V*_ 71.3 mV+−*E*_OHC_)/(EP_CBA/J_ 114.7 mV+−*E*_OHC_); *E*_OHC_=−50 mV (refs 3, 33, 34). Nonetheless, the maximal magnitudes of CM potentials in CD-1*Cx30*^*A88V/A88V*^ mice are similar to those of CBA/J mice. We have no good reason to assume changes in the number or function of OHCs involved in CM generation under these stimulus conditions. Thus, the finding of a preserved CM in spite of reduced transduction currents would indicate an increased electrical impedance of the cochlear partition in the mutant mice^33^. Indeed, a reduction in gap-junction conductance in CD-1*Cx30*^*A88V/A88V*^ has been demonstrated *in vitro*. Using electrophysiological analysis of paired *Xenopus* oocytes, Teubner and colleagues^46^ reported that the junctional conductance was smaller in cells expressing A88V Cx30 than in those connected by WT Cx30. The channels made by A88V Cx30 differed from those made by the WT form in their voltage gating properties^46^. It was proposed^46^ that the lower conductance values recorded in homotypic A88V pairs could be due to either or both of two mechanisms: (i) a reduced inter-connexon affinity in homotypic configuration, which results in a poor efficiency in channel formation and/or, (ii) altered intrinsic channel properties such as favouring a closed state in the absence of a transjunctional potential, reducing the open time probability and/or unitary conductance. Our hypothesis remains tentative until it is discovered how exactly the conductance properties of gap junctions expressing mutated Cx30 A88V connexins in the cochlea are changed and how this affects the electrical impedance of the cochlear partition in CD-1*Cx30*^*A88V/A88V*^ and CD-1*Cx30*^*A88V/WT*^ mice. In addition, the *Cx30*^*A88V/A88V*^ mice may also become a useful mouse model to study the role for Cx30 in the generation of the EP. It is known that genetic disruption of the gene coding for Cx30 leads to disruption of the tight-junctional networks of the SV and elimination of the EP^47^,^48^. However, such studies on knockout mice are complicated by the genetic interactions between the Cx26 and Cx30 genes^49^, and the possibility of compensation for the complete absence of Cx30 by overexpression of Cx26 (ref. 50).

As EP is reduced, the MET currents in individual OHC of CD-1*Cx30*^*A88V/A88V*^ mutants must be reduced not only at high but also at low stimulus intensities. To explain the preserved cochlear sensitivity, we suggest the predominant factor controlling OHC electromotility is not a change in the OHC intracellular potential resulting from the changing current flux through the OHC MET conductance^32^. Instead, our data support the proposal that voltage-dependent amplification is controlled by the OHC transmembrane potential changes which are due predominantly to changes in the OC potentials extracellular to the OHCs^14^,^51^,^52^. In this sense, the extracellular potentials in the vicinity of the OHCs provide ‘a floating ground' for the OHC transmembrane potential. These potentials^14^ are generated by the flow of sound-induced MET currents along their return pathways through the electrical impedance of the cochlear partition^33^,^53^, which we tentatively propose is increased in CD-1*Cx30*^*A88V/A88V*^ mice. Control of somatic motility by extracellular OC potentials would also enable the bandwidth of cochlear amplification to be limited only by that of the voltage-dependent motility itself^54^.

Thresholds in the low-frequency tails of the BM tuning curves are reduced in CD-1*Cx30*^*A88V/A88V*^ mice that may indicate a decrease in the apparent stiffness component of the complex mechanical impedance of the cochlear partition at these frequencies that would need to be confirmed in future direct measurements. The threshold reduction is not associated with observable phase changes. This may not be surprising because a reduction in stiffness of a system is associated not with phase changes but only with amplitude changes in the frequency range where the responses of the system are dominated by stiffness rather than by active nonlinear amplification, as it is the case in the low-frequency tails of the frequency tuning curves^22^. We tentatively propose that a common factor may be responsible for the enhanced BM frequency tuning of CD-1*Cx30*^*A88V/A88V*^ mice compared with CBA/J and other sensitive wild-type mice^39^,^40^ and for the reduced CM magnitude in response to low-intensity low-frequency tones. This proposed factor is a decrease in mechanical coupling within the cochlear partition due to the *Cx30 A88V* mutation. A similar change in the longitudinal mechanical properties of elements of the cochlear partition has previously been shown to sharpen the mechanical tuning of the cochlea in *Tectb*^*−/−*^ mice where the number of OHCs contributing towards amplification at a given cochlear location is reduced compared with that in control mice^40^. Here that element is the extracellular matrix of the TM and the change in its mechanical properties was attributed to loss of elastic coupling along its length^55^ due to the loss of the major TM protein β-tectorin^56^,^57^.

Indeed, gap junctions, the targets of the *Cx30 A88V* mutation, have previously been suggested to influence the mechanical properties of the cochlear partition^58^,^59^. It has been shown that gap junctions are mechanically sensitive in the inner ear^60^ and that their disruption impairs cochlear amplification^61^. It is proposed that changes in the properties of the mutated gap junctions could directly or indirectly provide a means for a change in longitudinal and perhaps radial coupling in the cochlear partition as a consequence of the *Cx30 A88V* mutation.

Similarly, reduced mechanical coupling within the cochlear partition with consequent smaller spread of excitation, can account for CM differences between CD-1*Cx30*^*A88V/A88V*^ and CBA/J mice at stimulation levels <60 dB SPL. It has previously been proposed that the CM, which reflects the total MET current generated in the basal turn in response to low-frequency tones, increases linearly with stimulus intensity as a consequence of (i) increased flow of MET current through each OHC and (ii) increased spread of excitation along the cochlear partition^36^. From our findings we propose that for stimulus levels <60 dB SPL), the spread of excitation and consequent recruitment of OHCs is more restricted in CD-1*Cx30*^*A88V/A88V*^ mice than in the CBA/J mice because of a reduction in mechanical coupling within the basal cochlear partition. Only when the tone level exceeds ∼60 dB SPL would more of the BM be recruited by the 5 kHz tone when the entire basal turn OHCs contribute to generation of the CM recorded at the RW.

Our *in vivo* data describing the effects of the *A88V* mutation of *Cx30* provides indirect evidence for new potential roles for gap junctions in sensory processing in the cochlea. Further *in vivo* and *in vitro* measurements are required to understand how the mutation influences the electrical and mechanosensitive properties of cochlear gap junction and how this alters the complex electrical environment of OHCs, thereby enabling them to contribute fully in their sensory-motor role to the sensitivity of the cochlea, how gap junctions contribute to the static and dynamic mechanical properties of the cochlear partition, and finally how the mutation rescues hearing in a mouse line that normally expresses accelerated ARHL.

## Methods

### Animals

Homozygous *Cx30*^*A88V/A88V*^ mice from a colony generated and supplied to us by Bosen *et al*.^13^ formed the basis for a new colony of *Cx30*^*A88V*^ mice maintained under quiet conditions in our facility. All experiments were performed with littermates, male and female, of >96.9% CD-1 background (>5 back crosses to the CD-1 background). CBA/J mice were obtained from Envigo.com, UK. All mice used in this study were kept under standard housing conditions with a 12 h/12 h dark–light cycle and with food and water *ad libitum*. Genotyping was performed according to the protocol provided by Bosen *et al*.^13^. All procedures involving animals were performed in accordance with the UK Home Office regulations with approval from the University of Brighton Animal Welfare and Ethical Review Body.

### Histological and immunofluorescence analyses

Isolated inner ear tissue was fixed with 4% paraformaldehyde in phosphate-buffered saline (PBS) for 2 h, then rinsed and frozen in Tissue-Tek embedding medium (Sakura, Zoeterwonde, The Netherlands), cryosectioned (∼10 μm), and immunostained with antibodies following standard protocols. The antibodies used were directed against rabbit anti-Cx30 (polyclonal, 1:250, Invitrogen, catalogue no. 71-2200). Immunostaining was visualized using Alexa Fluor 594 goat anti-rabbit IgG (1:1,000, Invitrogen, catalogue no. A-11037, CA, USA).

Omission of the primary antibodies eliminated staining in all preparations examined. The nucleus was counterstained with DAPI. A Leica confocal microscope was used to collect images. Leica LAS AF and Image-J software were used to collect and generate images.

### Physiological recordings

Mice, 3–5 weeks of age, were anaesthetized with ketamine (0.12 mg g^−1^ body weight i.p.) and xylazine (0.01 mg g^−1^ body weight i.p.) for nonsurgical procedures or with urethane (ethyl carbamate; 2 mg g^−1^ body weight i.p.) for surgical procedures. The animals were tracheotomized, and their core temperature was maintained at 38 °C. To measure BM displacements, CM (Fig. 2a), a caudal opening was made in the ventro-lateral aspect of the right bulla to reveal the RW. CM potentials were measured from the RW membrane by using glass pipettes filled with artificial perilymph, with tip diameters of 50–100 μm (recording bandwidth >30 kHz). Signals were amplified with a recording bandwidth of d.c. to 100 kHz using a laboratory designed and constructed preamplifier. With low-impedance electrodes, CM was measured at levels of 20 dB SPL in response to 5 kHz tones in mice with DPOAE responses that were sensitive throughout the 1–70 kHz range of the sound system. Intracellular electrodes (70–100 MΩ, 3 M KCl filled) were pulled from 1 mm O.D., 0.7 mm I.D. quartz glass tubing on a Sutter P-2000 micropipette puller (Sutter Instrument Novato, CA, USA). Signals were amplified and conditioned using laboratory built pre-amplifiers and conditioning amplifiers. Electrodes were advanced using a piezo-activated micropositioner (Marzhause GMBH). The pipette tip was inserted through the RW membrane and into the BM, close to the feet of the OPCs, under visual control. The first cells to be encountered had resting potentials of less than −80 mV, could be held for 10 s of minutes and were assumed to be supporting cells. Other cells encountered immediately before penetrating the scala media had resting potentials of approximately −50 mV and could be held for seconds to several minutes. These were presumed OHCs. Loss in sensitivity of the preparation was determined by changes in CM threshold. Losses were never encountered as a consequence of intracellular penetration with the electrode. Experiments were terminated immediately there was any loss in CM threshold (≥5 dB SPL) due usually to change in the condition of the preparation.

Sound was delivered via a probe with its tip within 1 mm of the tympanic membrane and coupled to a closed acoustic system comprising two MicroTechGefell GmbH 1-inch MK102 microphones for delivering tones and a Bruel and Kjaer ( www.Bksv.co.uk) 3135 0.25-inch microphone for monitoring sound pressure at the tympanum. The sound system was calibrated *in situ* for frequencies between 1 and 70 kHz by using a laboratory designed and constructed measuring amplifier, and known sound pressure levels (SPLs) were expressed in dB SPL with reference to 2 × 10^−5^ Pa. Tone pulses with rise/fall times of 1 ms were synthesized by a Data Translation 3010 (Data Translation, Marlboro, MA) data acquisition board, attenuated, and used for sound-system calibration and the measurement of electrical and acoustical cochlear responses. To measure DPOAEs, primary tones were set to generate 2f1–f2 distortion products at frequencies between 1 and 50 kHz. DPOAEs were measured for levels of f1 ranging from 10 to 80 dB SPL, with the levels of the f2 tone set 10 dB SPL below that of the f1 tone. DPOAE threshold curves were constructed from measurements of the level of the f2 tone that produced a 2f1–f2 DPOAE with a level of 0 dB SPL where the frequency ratio of f2:f1 was 1.23. System distortion during DPOAE measurements was 80 dB below the primary tone levels. Tone-evoked BM displacements were measured by focusing the beam of a self-mixing, laser-diode interferometer^62^ through the RW membrane to form a 20-μm spot on the centre of the basilar membrane in the 50–56 kHz region of the cochlea. The interferometer was calibrated at each measurement location by vibrating the piezo stack on which it was mounted over a known range of displacements. At the beginning of each set of BM measurements it was ensured that the 0.2 nm threshold used as the criterion for threshold was at least as sensitive as the 0 dB SPL threshold for the DPOAEs before the cochlea was exposed. BM measurements were checked continuously for changes in the sensitivity of the measurement (due to changes in alignment or fluid on the RW) and for changes in the condition of the preparation. If the thresholds of latter changed by more than 5–10 dB SPL, the measurements were terminated. Tone pulses with rise/fall times of 1 ms were used for the basilar membrane measurements. Stimulus delivery to the sound system and interferometer for calibration and processing of signals from the microphone amplifiers, microelectrode recording amplifiers, and interferometer were controlled by a DT3010/32 (Data Translation, Marlboro, MA) board by a PC running Matlab (The MathWorks, Natick, MA) at a sampling rate of 250 kHz. The output signal of the interferometer was processed using a digital phase-locking algorithm, and instantaneous amplitude and phase of the wave were recorded.

All measurements were performed blind. Measurements were made from each animal in a litter and data were analysed at the end of each set of measurements. When all measurements had been made from a particular litter, the tissue was genotyped. Randomization was not appropriate because we had no foreknowledge of the genotype, although we could guess it from the phenotype. Phenotypic differences between the WT, heterozygous and homozygous mice were very strong. Thus only sufficient numbers of measurements were made to obtain statistically significant differences. Experiments were terminated (<5% of all measurements) if the physiological state of the preparation changed during a measurement and data from the measurement was excluded.

### Data availability

All relevant data are available from the authors.

## Additional information

**How to cite this article:** Lukashkina, V. A. *et al*. A connexin30 mutation rescues hearing and reveals roles for gap junctions in cochlear amplification and micromechanics. *Nat. Commun.* **8,** 14530 doi: 10.1038/ncomms14530 (2017).

**Publisher's note:** Springer Nature remains neutral with regard to jurisdictional claims in published maps and institutional affiliations.
