# Corrigendum: A connexin30 mutation rescues hearing and reveals roles for gap junctions in cochlear amplification and micromechanics

**DOI:** 10.1038/ncomms15094

**Published:** 2017-03-21

**Authors:** Victoria A. Lukashkina, Snezana Levic, Andrei N. Lukashkin, Nicola Strenzke, Ian J. Russell

Nature Communications 8: Article number: 14530; DOI: 10.1038/ncomms14530 (2017); Published 02 21 2017; Updated 03 21 2017

The financial support for this Article was not fully acknowledged. The Acknowledgements should have included the following:

The research was funded by a grant from the Medical Research Council (MR/N004299/1) and the German Research Foundation (DFG) through the priority programme 1608.

